# Lacrimispora sinapis sp. nov., isolated from pickled potherb mustard (Brassica juncea Coss.)

**DOI:** 10.1099/ijsem.0.006675

**Published:** 2025-02-07

**Authors:** Quanlu Ren, Danqi Wang, Jin Han, Zhenmin Liu, Zhengjun Wu

**Affiliations:** 1 State Key Laboratory of Dairy Biotechnology, Shanghai Engineering Research Center of Dairy Biotechnology, Dairy Research Institute, Bright Dairy & Food Co., Ltd., Shanghai 200436, PR China

**Keywords:** catalase, *Lacrimispora*, novel species, pickled potherb mustard

## Abstract

Strain JR3^T^ was isolated from Chinese pickled potherb mustard (*Brassica juncea* Coss.) purchased from a local market in Shanghai, China. A polyphasic approach including 16S rRNA gene sequence analysis, average nucleotide identity (ANI) analysis, digital DNA–DNA hybridization (dDDH), determination of G+C content and various phenotypic analyses was employed to characterize strain JR3^T^. The bacterium was rod-shaped, Gram-stain-positive, terminal round endospore-forming and catalase-positive. The strain could grow at a wide range of temperatures (20–45 °C) and pH values (6.0–8.0). Optimal growth of strain JR3^T^ occurred at 35–40 °C and a pH value of 7.0. The strain exhibited growth at salt (NaCl) concentrations of up to 3% (w/v). The G+C content of the genomic DNA was 44.0 mol%. The major fatty acids were C16 : 0, C19 : 0 c9, 10, summed feature 10 (C18 : 1 c11/t9/t6) and C18 : 1 c9. 16S rRNA gene sequencing revealed that strain JR3^T^ represents a member of the genus *Lacrimispora*, and it has higher sequence similarity with *Lacrimispora amygdalina* BR-10^T^ (=DSM 12857^T^) (98.72%), *Lacrimispora saccharolyticum* WM1^T^ (98.29%) and *Lacrimispora xylanolytica* sy1 (98.22%). The dDDH value for strain JR3^T^ and phylogenetically related species within the genus *Lacrimispora* ranged from 17.7% to 29.9%. The ANI of strain JR3^T^ with its closely related taxa was far lower than the threshold (95%−96%) used for species differentiation. Results of phylogenetic, physiological and phenotypic characterization confirmed that strain JR3^T^ represented a novel species within the genus *Lacrimispora*, for which the name *Lacrimispora sinapis* sp. nov. is proposed. The type strain is JR3^T^=CCTCC AB 2024044^T^=LMG 33655^T^.

## Introduction

*Lacrimispora* is a newly established genus transferred from the *Clostridium* XIVa cluster commonly containing Gram-stain-positive, spore-forming anaerobes, with the type species *Lacrimispora sphenoides* [1]. Species of the genus *Lacrimispora* have a potential impact on human and animal health [2,3]. Currently, the genus *Lacrimispora* includes ten validated species isolated from various habitats, including *L. sphenoides* from gangrenous war wounds [4], *Lacrimispora aerotolerans* from sheep rumina [5], *Lacrimispora algidixylanolytica* from raw lamb [6], *Lacrimispora amygdalina* from a laboratory reactor treating potato starch wastewater [7], *Lacrimispora celerecrescens* from cow manure inoculum [8], *Lacrimispora indolis* from a patient operated for cancer in the large intestine [4], *Lacrimispora saccharolytica* from sewage sludge [9], *Lacrimispora xylanolytica* from pinus chips [10], *Lacrimispora xylanisolvens* from cattle manure [11,12] and *Lacrimispora brassicae* from fermented cabbage [13].

Pickling is one of the oldest methods to preserve foodstuffs, and microbial fermentation plays a pivotal role in the pickling of foodstuffs [14]. Traditionally, the homemade Chinese pickled potherb mustard (*Brassica juncea* Coss.) is wilted in the sun to collect microbes naturally and fermented for a couple of weeks [15]. In the present study, a novel bacterium, JR3^T^, was isolated from Chinese pickled potherb mustard and characterized by a polyphasic approach.

## Isolation and ecology

Pickled potherb mustard was collected from a local agriculture products market in Hongkou, Shanghai, China (latitude 31.27° N, longitude 121.49° E). The pickle sample was chopped into pieces with 3–4 mm long and soaked in saline for 30–35 min. The bacterial suspension was diluted by tenfold series and spread on DeMan-Rogosa-Sharpe (MRS) agar. The plates were incubated at 36 °C in a Bugbox (Baker Ruskinn, England) with an O_2_-free environment comprising N2/H2/CO_2_ (80%/10%/10%) for 3 days. The colonies with around agar swelled for gas production on MRS agar were picked for further study.

## 16S rRNA gene phylogeny

**Fig. 1.**
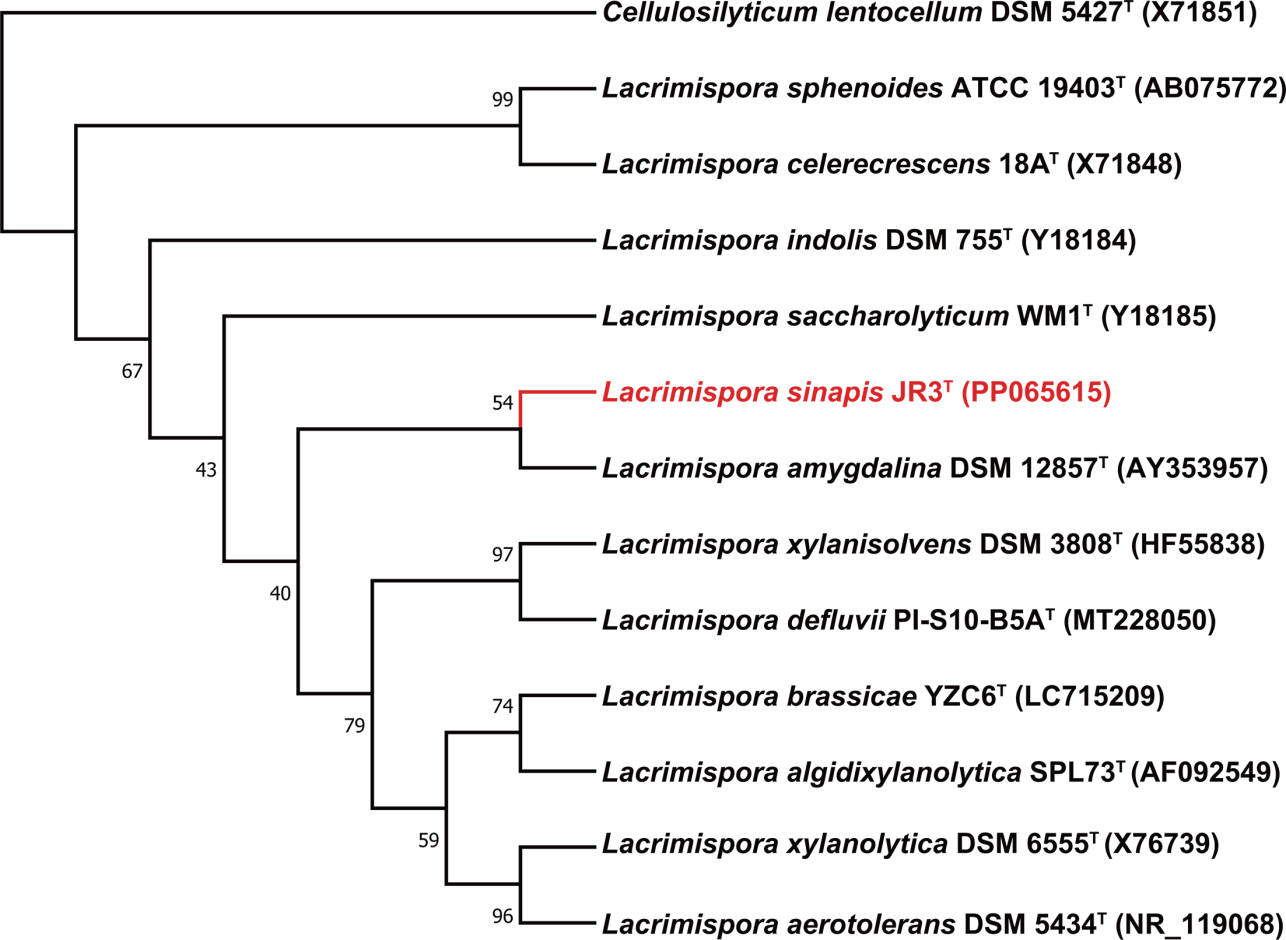
Phylogenetic tree of 16S rRNA genes.

The primers 27F and 1492R were utilized to amplify the 16S rRNA gene of strain JR3^T^ via PCR; the amplicon was subsequently sequenced to obtain the 16S rRNA gene sequence. The sequence data (1403 bp) were compared with 16S rRNA gene sequences available in the GenBank databases using the blast tool. Based on the blasting result, the 16S rRNA sequences of type strains of species in the genus *Lacrimispora* as well as strain JR3^T^ were selected to construct the phylogenetic tree by maximum likelihood method by mega 6.0 software [16]. In the phylogenetic tree, strain JR3^T^ formed an individual cluster, apart from the coherent cluster formed by the type strains of *L. amygdalina* (Fig. 1), which confirmed that strain JR3^T^ was included in the *Lacrimispora* clade. The 16S rRNA gene sequence of strain JR3^T^ showed the highest similarity to *L. amygdalina* BR-10^T^ (=DSM 12857^T^) (98.72%), *L. saccharolyticum* WM1^T^ (98.29%) and *L. xylanolytica* sy1 (98.22%).

## Genome features

Strain JR3^T^ was streaked on MRS agar and cultured at 36 °C for 72 h. Subsequently, a single colony was inoculated into 10 ml of MRS broth and cultured statically at 36 °C for ~24 h. The cell biomass was harvested by centrifugation at 4 °C, 10 000 ***g*** for 15 min. The genomic DNA of strain JR3^T^ was extracted using Wizard® Genomic DNA Purification Kit (Promega) according to the manufacturer’s protocol. Purified genomic DNA was quantified by TBS-380 fluorometer (Turner BioSystems Inc., Sunnyvale, CA). High-quality DNA (OD260/280=1.8~2.0, ≥10 µg) was employed for the sequencing.

Prior to the sequencing of the whole genome, the purity of the extracted genomic DNA was checked by 16S rRNA encoding gene amplification utilizing the universal primers. Genomic DNA was sequenced using a combination of PacBio Sequel II and Illumina sequencing platforms. The Illumina data were used to evaluate the complexity of the genome. For Illumina sequencing, DNA samples were sheared into 400–500 bp fragments using a Covaris M220 Focused Acoustic Shearer following the manufacturer’s protocol. Illumina sequencing libraries were prepared from the sheared fragments using the NEXTflex™ Rapid DNA-Seq Kit. The prepared libraries were then used for paired-end Illumina sequencing (2×150 bp) on an Illumina NovaSeq 6000 machine. For PacBio sequencing, DNA fragments were purified, end-repaired and ligated with SMRT bell sequencing adapters following manufacturer’s recommendations (Pacific Biosciences, CA). The resulting sequencing library was purified three times using 0.45× volumes of Agencourt AMPure XP beads (Beckman Coulter Genomics, MA) following the manufacturer’s recommendations. Next, a ~10 kb insert library was prepared and sequenced on one SMRT cell using standard methods. The data generated from the PacBio and Illumina platforms were analysed using the free online platform of Majorbio Cloud Platform (http://cloud.majorbio.com) [17,19]. The raw Illumina sequencing reads generated from the paired-end library were subjected to quality filtering using fastp v0.23.0. The raw sequencing reads generated from the PacBio platform were processed using SMRT Analysis v2.3.0. Then, the clean short and long reads were coassembled to construct complete genomes using Unicycler v0.4.8. As a final step, Wizard® Genomic DNA Purification Kit (Promega) Unicycler uses Pilon v1.22 to polish the assembly using short-read alignments, reducing the rate of small errors. The CDs of chromosome were predicted using Prodigal v2.6.3. tRNA-scan-SE (v 2.0) for tRNA prediction and Barrnap (v 0.9) for rRNA prediction. The predicted CDs were annotated from NR, Swiss-Prot, Pfam, GO, Clusters of Orthologous Group (COG) and KEGG databases using sequence alignment tools (blast+). Briefly, each set of query proteins was aligned with the databases, and annotations of best-matched subjects (e-value <10^−5^) were obtained for gene annotation. KEGG pathway classification and proportions of genes associated with each COG category are shown in Figs S1 and S2 (available in the online Supplementary Material).

The genome of strain JR3^T^ comprised one contig with a total length of 3 753 647 bp. It had a total DNA G+C content of 44.03 mol%. Strain JR3^T^ has the smallest genome size so far in the genus (Table S1). Whole-genome sequencing revealed a total of 3439 genes, 65 tRNAs and 18 rRNAs. Circular genomic representation is shown in Fig. S3. In the genome of strain JR3^T^, four catalase (CAT) genes were annotated, and the CAT activity of strain JR3^T^ was detected in the CAT test. No CAT activity was detected in *L. amygdalina* BR-10^T^ (=DSM 12857^T^) or *L. xylanolytica* ATCC 49623^T^. Furthermore, in the genome of strain *L. amygdalina* BR-10^T^ (=DSM 12857^T^), no CAT gene was annotated, whilst the genome of strain *L. xylanolytica* ATCC 49623^T^ is not available in NCBI databases. In addition, CAT activity and genome annotations were searched in other species in the genus *Lacrimispora*. No CAT activity was reported in the genus *Lacrimispora*, but CAT family protein was annotated in the genomes of all species in the genus *Lacrimispora* except *L. amygdalina* BR-10^T^ (=DSM 12857^T^) and *L. brassicae* YZC6^T^, as shown in Table S2. CAT was ubiquitously present in mammalian and nonmammalian aerobic cells containing a cytochrome system [20], to protect against the toxic and mutagenic compounds from the reduction of oxygen. The clinical features of acatalasemia revealed a high incidence in childhood, a shortened lifespan and also the correlation with various cancers in adult persons [21]. In future work, the four CAT genes in strain JR3^T^ are worthy of exploring for their role in bacteria cells to adoption of individual niche.

**Fig. 2.**
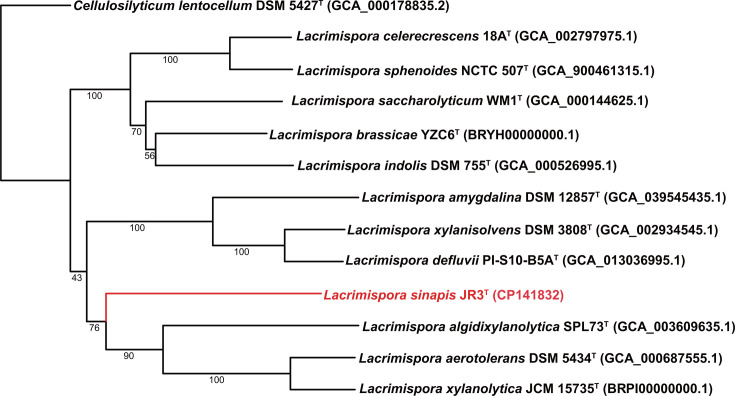
Phylogenetic tree based on whole-genome sequences.

The genome sequence of JR3^T^ was uploaded to the Type (Strain) Genome Server (TYGS), a free bioinformatics platform available under https://tygs.dsmz.de, for whole genome-based taxonomic analysis [22]. The analysis also adapted recently introduced methodological updates and features [23]. For the phylogenomic inference, all pairwise comparisons among the set of genomes were conducted using Genome blast Distance Phylogeny, and accurate intergenomic distances were inferred under the algorithm 'trimming' and distance formula *d*_5_ [24]. One hundred distance replicates were calculated each. Digital DNA–DNA hybridization (dDDH) values and CIs were calculated using the recommended settings of the GGDC 4.0 [23,24]. The resulting intergenomic distances were used to infer a balanced minimum evolution tree with branch support via FASTME 2.1.6.1 including Subtree Pruning and Regrafting topological moves [25]. Branch support was inferred from 100 pseudo-bootstrap replicates each. The trees were rerooted at the outgroup and visualized with PhyD3 [26]. As shown in Fig. 2, the evolution tree confirmed that strain JR3^T^ was within the genus of *Lacrimispora* and apart from other species.

To determine its exact taxonomic status, the average nucleotide identity calculated based on blast (ANIb) and dDDH values of the whole genome of strain JR3^T^ were compared with other members in the genus *Lacrimispora* using JSpeciesWS [27] and TYGS [23,24], respectively. The ANIb and dDDH ranges for related type strains were 75.11%–78.58% and 17.7%–29.9%, respectively (Fig. S4, Table 1). These values were lower than the cutoff values recommended for species delineation, which are 95%−96% (ANIb) and 70% (dDDH). When combined with the low 16S rRNA gene sequence similarities, these genomic data indicate that strain JR3^T^ represents a novel species in the genus *Lacrimispora*.

**Table 1.** Identities of 16S RNA gene sequence, ANIb of whole genome and dDDH of *L. sinapis* JR3^T^ vs. species within the genus *Lacrimispora*. CIs of dDDH are provided

*L. sinapis* JR3^T^ vs. reference strain	Accession nos.	16S rDNA (%)	ANIb (%)	Aligned (%)	dDDH (%)	Model CI
*L. xylanolytica* JCM 15735^T^	BRPI00000000.1	97.29	78.58	67.49	29.9	(26.5–33.5%)
*L. aerotolerans* DSM 5434^T^	GCA_000687555.1	97.65	78.45	67.66	28.3	(25–32%)
*L. algidixylanolytica* SPL73^T^	GCA_003609635.1	97.86	77.79	67.61	26.5	(23.2–30.2%)
*L. amygdalina* BR-10^T^=DSM 12857^T^	GCA_039545435.1	98.72	75.11	59.1	19	(15.9–22.6%)
*L. xylanisolvens* DSM 3808^T^	GCA_002934545.1	97.93	75.21	60.44	19	(15.9–22.6%)
*L. saccharolyticum* WM1^T^	GCA_000144625.1	98.29	76.98	48.28	18.5	(15.4–22.1%)
*L. sphenoides* NCTC507^T^	GCA_900461315.1	97.51	76.85	52.84	18.3	(15.2–21.9%)
*L. celerecrescens* 18A^T^	GCA_002797975.1	97.36	76.84	51.99	17.9	(14.9–21.5%)
*L. indolis* DSM 755^T^	GCA_000526995.1	97.29	76.76	55.19	17.8	(14.7–21.3%)
*L. brassicae* YZC6^T^	BRYH00000000.1	97.22	76.83	51.48	17.7	(14.6–21.2%)

## Physiology and chemotaxonomy

In the present study, *L. amygdalina* BR-10^T^=DSM 12857^T^ and *L. xylanolytica* ATCC 49623^T^, phylogenetically related to strain JR3^T^, were used for phenotypic and chemotaxonomic comparisons. After 72 h of anaerobic incubation on MRS agar at 36 °C, the colony and cell morphology of strain JR3^T^ were observed. The electron microscope images were obtained according to the manual instructions of the scanning electron microscope. Gram staining and spore staining were performed as described by Kamlage *et al*. and Bartholomew and Mittwer [28,29]. In order to determine the optimal growth conditions, strain JR3^T^ and reference strains (*L. amygdalina* BR-10^T^=DSM 12857^T^ and *L. xylanolytica* ATCC 49623^T^) were individually inoculated in MRS broth for 48 h at 5, 10, 15, 20, 25, 30, 35, 40, 45 and 50 °C; pH 3.0–10.0 at 1.0 pH unit intervals at 36 °C; and 0–8% NaCl (w/v) with increments of 1% at 36 °C. Acid production from carbon sources and enzyme activity was tested by using API 50CH, API 20A and API ZYM strips according to the manufacturer’s instructions (bioMérieux).

Cells of strain JR3^T^ grown in MRS broth to exponential phase were harvested for chemotaxonomic assays. Cellular fatty acids were extracted, methylated and analysed using the Sherlock Microbial Identification System (MIDI) according to the manufacturer’s instructions. Polar lipids were extracted with chloroform/methanol and identified by two-dimensional TLC followed by spraying with the appropriate detection reagents [30]. Amino acids and sugars of cell wall were investigated according to the method described by Hasegawa *et al*. [31], with the minor modification that TLC on cellulose sheets was used instead of paper chromatography. Oxidase activity was determined by API 55635 kits (bioMérieux), and CAT activity was determined by bubble production in 3% (v/v) H_2_O_2_. Other physiological and biochemical tests, such as Tween 80 hydrolysis, were performed according to methods described previously by Battley [32].

**Fig. 3.**
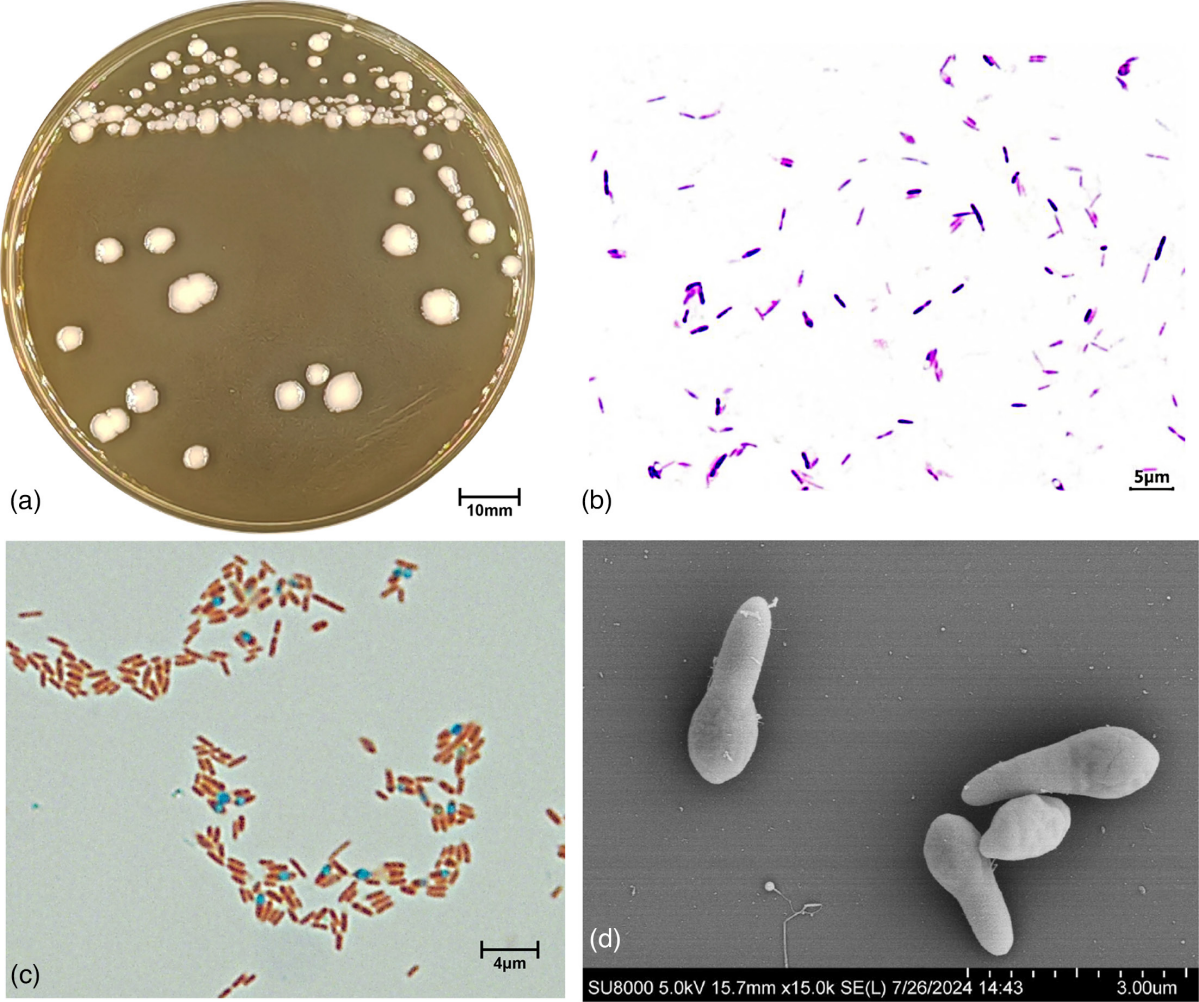
The colony formed by JR3^T^ on the MRS plate (**a**), Gram staining (**b**), spore staining (**c**) and scanning electron microscopy (**d**) of JR3^T^ cells.

Colonies formed by strain JR3^T^ on MRS agar at 36 °C for 3 days were up to 3–6 mm in diameter and pale yellow, nearly round, with irregular edges and glossy in appearance (Fig. 3a). Cells were round rods, 0.5–0.7 µm in width and 1.0–3.2 µm in length; spores were oval, terminal or subterminal; and the cysts were swollen (Fig. 3b, c). Growth occurred at a temperature of 20–45 °C, pH 6.0–8.0 and a NaCl concentration of 0–3% (w/v). The carbon source utilization of strain JR3^T^ is shown in Table 2. Strain JR3^T^ could be distinguished through inulin, d-melezitose, d-raffinose, starch, glycogen and d-toulose. API 20A and API ZYM assay results are also shown in Table 2. Urease and indole are negative. Worthy of note, strain JR3^T^ was CAT-positive, and the sole in the genus *Lacrimispora*.

**Table 2.** Physio-chemical characteristics of *L. sinapis* JR3^T^, *L. amygdalina* BR-10^T^ (=DSM 12857^T^) and *L. xylanolytica* ATCC 49623^T^

	*L. sinapis* JR3^T^	*L. amygdalina* BR-10^T^=DSM 12857^T^	*L. xylanolytica* ATCC 49623^T^
Growth			
Temperature (°C)	35–40	35–40	35
20 ℃	+	+	+
25 ℃	+	+	+
30 ℃	+	+	+
35 ℃	++	++	++
40 ℃	++	++	+
45 ℃	+	+	−
Growth pH	7.0	7.0	7.0
pH 6.0	+	+	−
pH 7.0	++	++	++
pH 8.0	+	+	+
Growth NaCl (%)	0–3	0–2	0–2
0% NaCl	++	++	++
1% NaCl	++	++	++
2% NaCl	+	+	+
3% NaCl	+	−	−
Acid production from (API 50 CH)			
l-Arabinose	+	+	+
d-Ribose	−	+	−
d-Xylose	+	+	+
d-Galactose	+	+	+
d-Glucose	+	+	+
d-Fructose	+	+	+
d-Mannose	+	+	+
l-Rhamnose	+	+	+
α-Methyl-d-mannoside	+	−	−
α-Methyl-d-glucoside	−	+	+
*N*-Acetylglucosamine	−	−	w
Amygdalin	+	+	w
Arbutin	+	−	+
Aesculin	+	+	+
Salicin	+	+	+
d-Cellobiose	+	+	+
d-Maltose	−	+	+
d-Lactose	+	+	+
d-Melibiose	+	+	+
d-Sucrose	+	+	+
d-Trehalose	−	w	+
Inulin	+	−	−
d-Melezitose	−	+	+
d-Raffinose	−	+	+
Starch	−	+	+
Glycogen	−	+	+
d-Gentiobiose	+	+	+
d-Toulose	−	+	+
d-Tagatose	−	−	+
2-Keto-gluconate	+	−	+
Activity of (API 20A)			
Arginine dihydrolase	−	+	−
Lysine decarboxylase	−	−	−
Ornithine decarboxylase	−	−	−
Citrate utilization	−	w	−
H_2_S production	−	+	−
Urease	−	−	−
Tryptophan deaminase	−	−	−
Indole production	−	−	−
VP test	−	−	−
Gelatin hydrolysis	−	+	−
Nitrate reduction	−	−	−
Arginine dihydrolase	−	+	−
Activity of (API ZYM)			
Alkaline phosphatase	+	+	+
Acid phosphatase	+	+	+
Esterase (C4)	+	+	+
Lipid esterase (C8)	+	+	+
Esterase (C14)	−	−	−
Leucine arylaminease	−	−	−
Valine arylaminease	−	−	−
Cystine arylaminease	−	−	−
Trypsin	−	+	−
Chymotrypsin	−	+	−
Naphthol-AS-BI-phosphohydrolase	+	+	+
α-Galactosidase	w	+	+
β-Galactosidase	+	+	+
β-Uronidase	−	+	+
β-Glucosidase	+	+	+
Other tests			
Oxidase	−	−	−
CAT	+	−	−
Casein hydrolysis	−	+	−
Tween 80	+	−	+

Notes: +, positive/growth; −, negative/no growth; ++, best growth; w, weak positive.

In the fatty acid profiles of strain JR3^T^, fatty acids C16 : 0 (19.6%), C19 : 0 c9, 10 (19.1%), summed feature 10 (C18 : 1 c11/t9/t6) (13.0%) and C18 : 1 c9 (10.2%) were the major components. The main components of polar lipids were unidentified amino phospholipid, unidentified phospholipids 1-4 and unidentified lipids 1-13. Cell wall characteristic sugars were composed of ribose and glucose, and diaminopimelic acid (DAP) was meso-DAP. Benzaldehyde reduction was performed referring to the method of Parshina *et al.* [33]. Strain JR3^T^ exhibits weak benzaldehyde reduction ability (Fig. S5). Based on the results of phenotypic, chemotaxonomic, phylogenetic and genomic assays, strain JR3^T^ represented a novel species within the genus *Lacrimispora*, for which the name *Lacrimispora sinapis* sp. nov. is proposed.

## Description of *Lacrimispora sinapis* sp. nov.

*Lacrimispora sinapis* (si.na’pis. L. gen. n. *sinapis*, of mustard).

Cells are rod-shaped, Gram-stain-positive, terminal round endospore-forming and CAT-positive. Growth occurs anaerobically. Cells are 0.5–0.7 µm in width and 1.0–3.2 µm in length. Colonies formed on MRS agar at 36 °C for 3 days under anaerobic conditions are up to 3–6 mm in diameter and pale yellow, nearly round, with irregular edges and glossy in appearance. In the API 50 CH test system, acids are produced from l-arabinose, d-xylose, d-galactose, d-glucose, d-fructose, d-mannose, l-rhamnose, α-methyl-d-mannoside, amygdalin, arbutin, aesculin, salicin, d-cellobiose, d-lactose, d-melibiose, d-sucrose, inulin, d-gentiobiose and 2-keto-gluconate but not from others. Growth occurs at a temperature of 20–45 °C, pH 6.0–8.0 and a NaCl concentration of 0–3% (w/v). C16 : 0, C19 : 0 c9, 10, summed feature 10 (C18 : 1 c11/t9/t6) and C18 : 1 c9 are the major fatty acids. The cell wall characteristic sugars are composed of ribose and glucose, and DAP is meso-DAP.

The type strain JR3^T^ (=CCTCC AB 2024044^T^=LMG 33655^T^) was isolated from Chinese pickled potherb mustard. The genomic DNA G+C content of strain JR3^T^ is 44.0 mol%. The GenBank accession number for the 16S rRNA gene sequence of strain JR3^T^ is PP065615 (https://www.ncbi.nlm.nih.gov/nuccore/PP065615) and for the genome sequence of strain JR3^T^ is CP141832 (https://www.ncbi.nlm.nih.gov/nuccore/ CP141832.1).

## supplementary material

10.1099/ijsem.0.006675 Uncited Supplementary Material 1.

## Abbreviations

ANI: average nucleotide identity
ANIb: average nucleotide identity calculated based on blast
CAT: catalase
COG: Clusters of Orthologous Group
dDDH: digital DNA-DNA hybridization
MRS: DeMan-Rogosa-Sharpe
TYGS: Type (Strain) Genome Server
