# Supplementary material for: Lacrimispora sinapis sp. nov., isolated from pickled potherb mustard (Brassica juncea Coss.)
Source: Int J Syst Evol Microbiol. 2025 Feb 7;75(2):006675. doi: 10.1099/ijsem.0.006675 (PMC11806200; doi:10.1099/ijsem.0.006675)
Supplement: Uncited Supplementary Material 1. [file ijsem-75-06675-s001.pdf]

IJSEM supplementary materials for:

# *Lacrimispora sinapis* sp. nov., a new species isolated from pickled potherb mustard (*Brassica juncea* Coss.)

Quanlu Ren, Danqi Wang, Jin Han, Zhenmin Liu and Zhengjun Wu*

State Key Laboratory of Dairy Biotechnology, Shanghai Engineering Research Center of Dairy Biotechnology, Dairy Research Institute, Bright Dairy & Food Co., Ltd., Shanghai 200436, PR China

* Corresponding author: Zhengjun Wu, E-mail: wuzhengjun@brightdairy.com

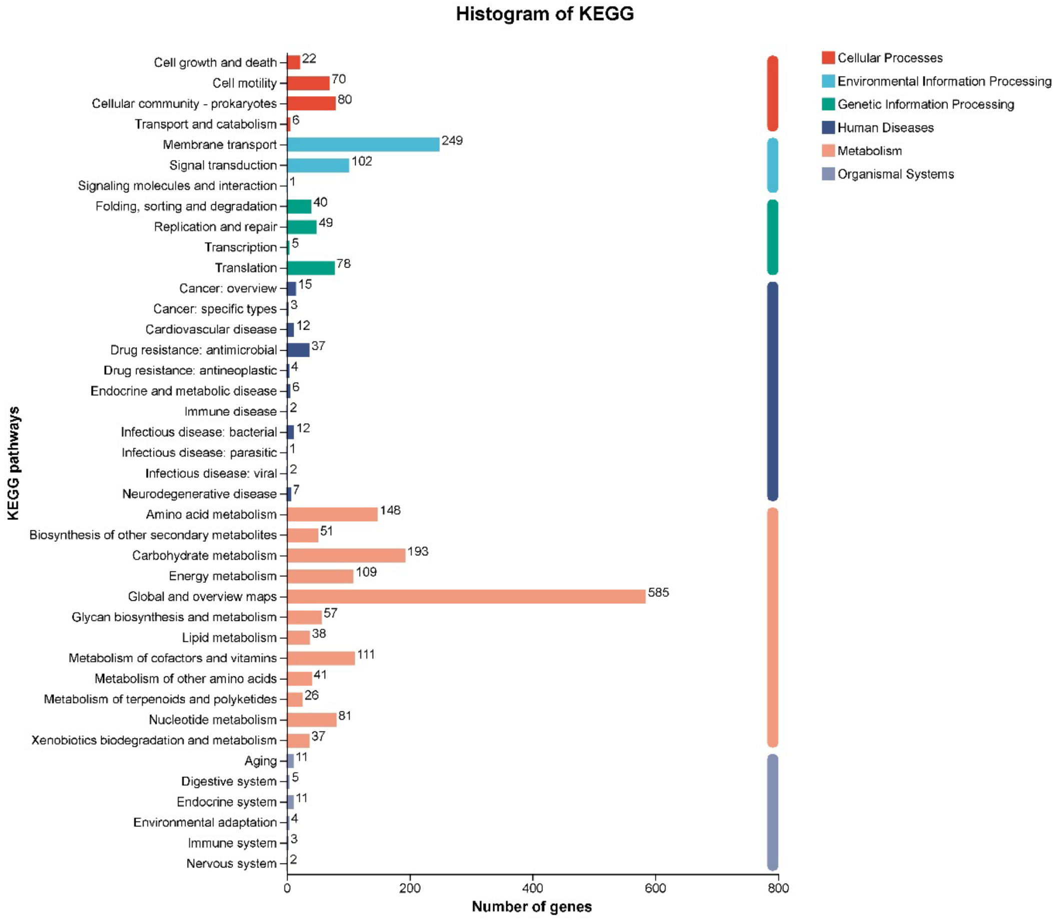


Fig. S1. KEGG pathway classification of *Lacrimispora sinapis* JR3$^{T}$. The vertical axis represents the level 2 classification of KEGG pathway, and the horizontal axis represents the number of genes annotated under this classification. Different column colors represent the level 1 classification of KEGG pathway.

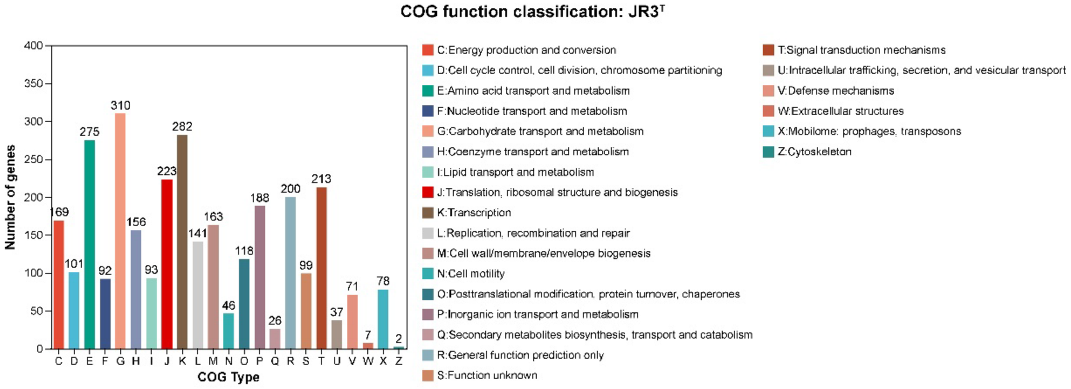


Fig. S2. Proportions of genes associated with each clusters of orthologous group (COG) category of *Lacrimispora sinapis* JR3$^T$

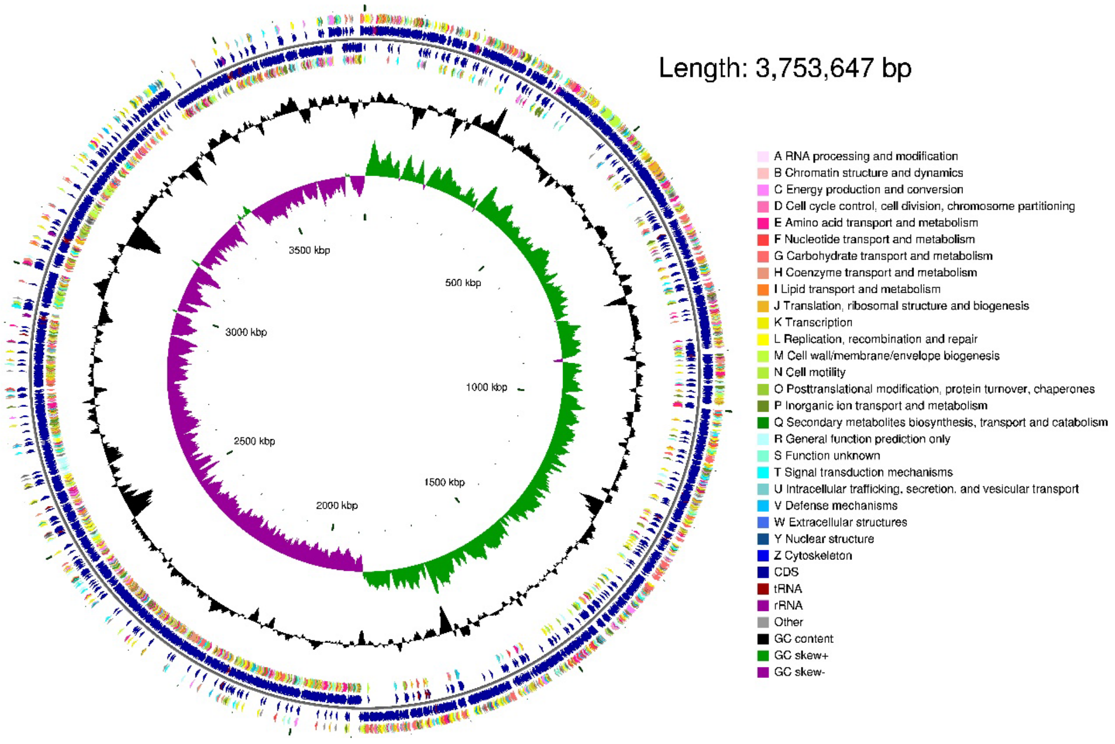


Fig. S3. Circular genomic representation of *Lacrimispora sinapis* JR3$^{T}$

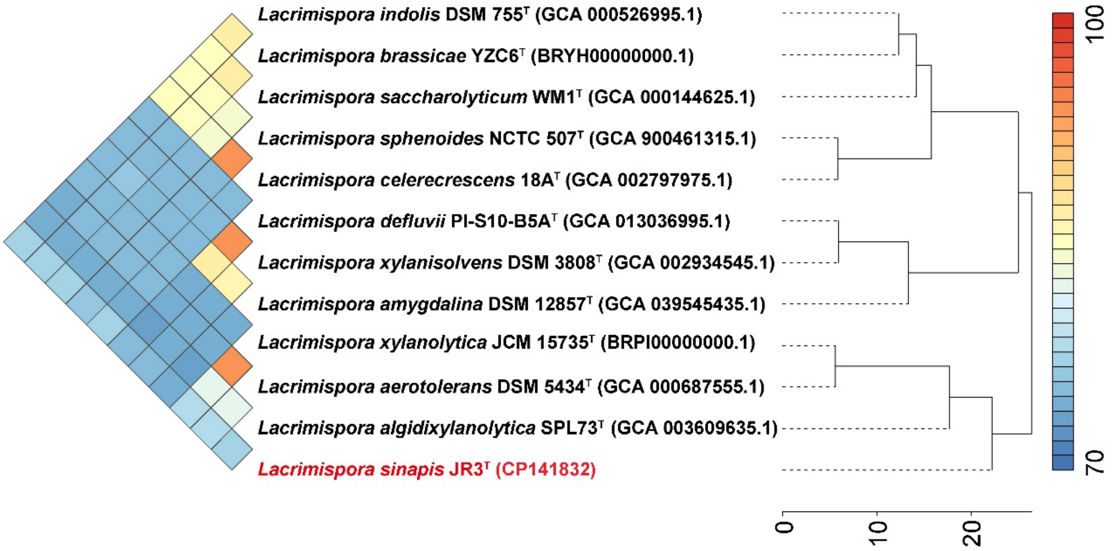


Fig. S4. Pairwise comparisons of average nucleotide identity (ANI) values between strain *Lacrimispora sinapis* JR3$^{T}$ and related taxa[1]. The color indicates the value of ANI. The genome distance is (1-ANI) %.

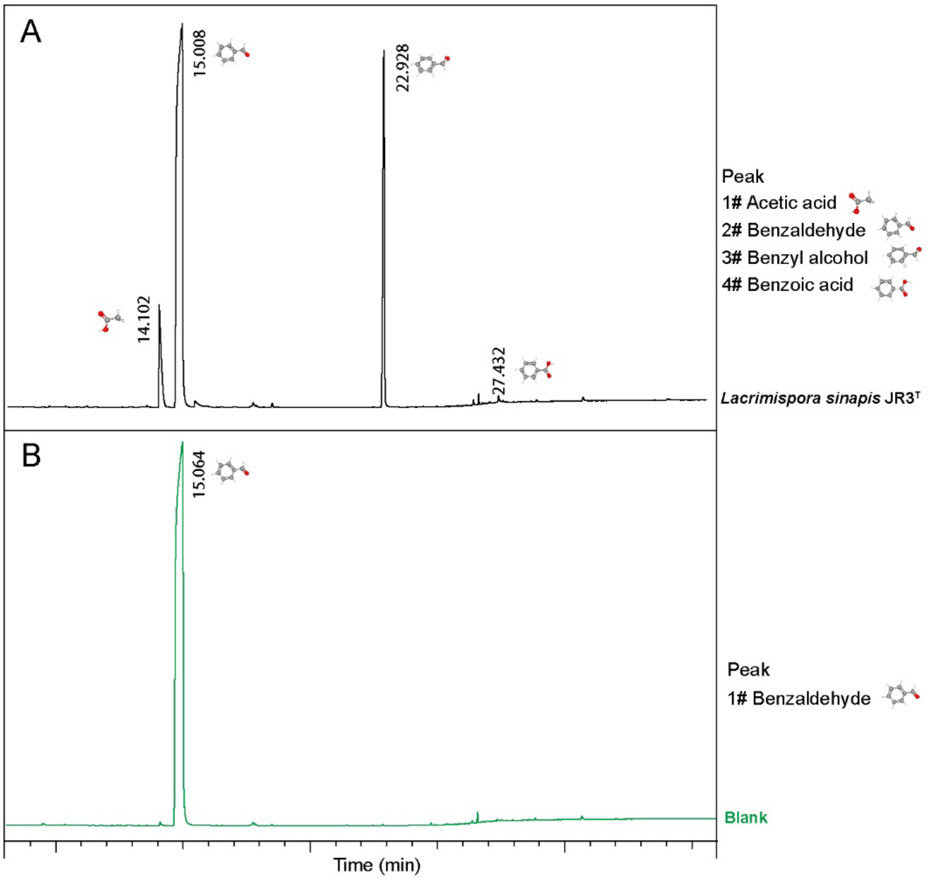


Fig. S5. Gas chromatography spectrum of benzaldehyde reduction of strain JR3$^T$ (A) and Blank (B). Benzaldehyde reduction was performed referring to the method of Parshina[2], strain JR3$^T$ was inoculated into a culture medium containing 10 mM benzaldehyde and incubated in anaerobic environment, collected the supernatant by centrifugation and did gas chromatography-mass spectrometry analysis. Thermo Scientific ISQ 7000 and TRACE 1300 equipped with Shimadzu SH-I-5Sil MS column (30m×0.25 mm×0.25 µm) was used. The applied temperatures were: 50 °C for 3 min and sequentially increased at the rate of 10 °C/min to 280 °C for 10 min, injection port 280 °C, and the carrier gas was helium, gas flow 1 mL/min. Mass spectrometry acquisition range 33-550 m/z. 20 % benzyl alcohol was detected, and there were trace amounts of benzoic acid.

Table S1. Genome characteristics and proteins number in the genus *Lacrimispora*.

| Genome | Base pairs | Percent G+C | No. proteins |
|---|---|---|---|
| *Lacrimispora sinapis* JR3$^T$ (CP141832) | 3,753,647 | 44.03 | 3448 |
| *Lacrimispora xylanolytica* JCM 15735$^T$ (BRPI00000000.1) | 4,620,800 | 41.89 | 4269 |
| *Lacrimispora brassicae* YZC6$^T$ (BRYH00000000.1) | 6,059,566 | 44.57 | 5448 |
| *Lacrimispora saccharolyticum* WM1$^T$ (GCA_000144625.1) | 4,662,871 | 45 | 4241 |
| *Lacrimispora indolis* DSM 755$^T$ (GCA_000526995.1) | 6,383,701 | 44.93 | 5711 |
| *Lacrimispora aerotolerans* DSM 5434$^T$(GCA_000687555.1) | 4,732,373 | 42.37 | 4300 |
| *Lacrimispora celerecrescens* 18A$^T$ (GCA_002797975.1) | 5,272,838 | 43.89 | 4692 |
| *Lacrimispora xylanisolvens* DSM 3808$^T$ (GCA_002934545.1) | 5,629,247 | 41.85 | 5279 |
| *Lacrimispora algidixylanolytica* SPL73$^T$ (GCA_003609635.1) | 4,639,933 | 40.22 | 4237 |
| *Lacrimispora defluvii* PI-S10-B5A$^T$ (GCA_013036995.1) | 5,624,842 | 42.39 | 5382 |
| *Lacrimispora amygdalina* DSM 12857$^T$ (GCA_039545435.1) | 5,350,244 | 42.29 | 5017 |
| *Lacrimispora sphenoides* NCTC 507$^T$ (GCA_900461315.1) | 5,309,474 | 43.78 | 4733 |

Table S2. Annotation of catalase family protein in the genomes of all species in the genus *Lacrimispora.*

| | genomic location | orientation | proteins | length(aa) |
|---|---|---|---|---|
| *Lacrimispora aerotolerans* DSM 5434 [T] | NZ_JHWJ01000007.1:71031-71651 | plus | WP_026891717.1 | 206 |
| *Lacrimispora algidixylanolytica* SPL73 [T] | NZ_MCIA01000023.1:71664-72290 | plus | WP_120197227.1 | 208 |
| | NZ_MCIA01000023.1:250625-251233 | minus | WP_120197379.1 | 202 |
| *Lacrimispora amygdalina* DSM 12857 [T] | —— | —— | —— | —— |
| *Lacrimispora brassicae* YZC6 [T] | —— | —— | —— | —— |
| *Lacrimispora celerecrescens* 18A [T] | NZ_PGET01000001.1:2737906-2738526 | plus | WP_100305468.1 | 206 |
| *Lacrimispora defluvii* PI-S10-B5A [T] | NZ_JAAOXG010000041.1:80279-80896 | plus | WP_170823009.1 | 205 |
| | NZ_JAAOXG010000044.1:1-526 | plus | WP_324293280.1 | 174 |
| *Lacrimispora indolis* DSM 755 [T] | NZ_AZUI01000001.1:5578649-5580166 | plus | WP_024295884.1 | 505 |
| | NZ_AZUI01000001.1:6265549-6266169 | plus | WP_024296488.1 | 206 |
| *Lacrimispora saccharolyticum* WM1 [T] | NC_014376.1:1384976-1385596 | minus | WP_013271981.1 | 206 |
| *Lacrimispora sphenoides* NCTC507 [T] | NZ_UFXF01000001.1:1555674-1556294 | minus | WP_038278883.1 | 206 |
| | NZ_UFXF01000001.1:3621767-3622375 | minus | WP_054791649.1 | 202 |
| *Lacrimispora xylanisolvens* DSM 3808 [T] | NZ_PTJA01000004.1:56413-56952 | minus | WP_104436766.1 | 179 |
| | NZ_PTJA01000005.1:143664-144281 | plus | WP_104436903.1 | 205 |
| *Lacrimispora xylanolytica* sy1 | NZ_CP113524.1:1352122-1352727 | plus | WP_268116048.1 | 201 |
| | NZ_CP113524.1:3066935-3067555 | plus | WP_024838336.1 | 206 |
| *Lacrimispora sinapis* JR3 [T] | NZ_CP141832.1:1151956-1152576 | minus | WP_013271981.1 | 206 |
| | NZ_CP141832.1:2545928-2546323 | minus | WP_002603012.1 | 167 |
| | NZ_CP141832.1:2555366-2555488 | minus | WP_008980832.1 | 212 |
| | NZ_CP141832.1:2558956-2559051 | minus | WP_015520420.1 | 202 |

## References


1 Liu, D. *et al.* IPGA: A handy integrated prokaryotes genome and pan-genome analysis web service. *iMeta* **1**, e55 (2022). https://doi.org:https://doi.org/10.1002/imt2.55

2 Parshina, S. N. *et al.* Benzaldehyde conversion by two anaerobic bacteria isolated from an upflow anaerobic sludge bed reactor. *Process Biochemistry* **36**, 423-429 (2000). https://doi.org:https://doi.org/10.1016/S0032-9592(00)00230-2
